# The Study of Myo-Inositol’s Anxiolytic Activity on Zebrafish (*Danio rerio*)

**DOI:** 10.3390/nu16131997

**Published:** 2024-06-23

**Authors:** Maria Derkaczew, Bartosz Kędziora, Małgorzata Potoczna, Piotr Podlasz, Krzysztof Wąsowicz, Marcin Jóźwik, Joanna Wojtkiewicz

**Affiliations:** 1 Department of Human Physiology and Pathophysiology, School of Medicine, Collegium Medicum, University of Warmia and Mazury, 10-082 Olsztyn, Poland; 2 Students’ Scientific Club of Pathophysiologists, Department of Human Physiology and Pathophysiology, School of Medicine, University of Warmia and Mazury, 10-082 Olsztyn, Poland; 3 Department of Pathophysiology, Forensic Veterinary Medicine and Administration, Faculty of Veterinary Medicine, University of Warmia and Mazury, 10-719 Olsztyn, Poland; malgorzata.mierzejewska@transpharmation.co.uk (M.P.); krzysztof.wasowicz@uwm.edu.pl (K.W.); 4 Department of Gynecology and Obstetrics, Collegium Medicum, University of Warmia and Mazury, 10-045 Olsztyn, Poland

**Keywords:** myo-Inositol, anxiety, anxiolytics, zebrafish

## Abstract

Introduction: Myo-inositol (MI) is the most abundant inositol found in nature. To date MI supplementation is reported to be effective in the treatment of polycystic ovary syndrome, it is also suggested to alleviate the symptoms of diabetes and neurodegenerative disorders, but to date no statistically significant effects of inositol on depressive and anxiety symptoms were proven. In the study of anxiolytic effects in zebrafish, we often use the thigmotaxis index measuring the ratio of the amount of time the animal spends near the walls compared to the entire arena. Aim: The objective of this paper was to examine the effect of MI on zebrafish embryos’ locomotor activity, as well as its potential anxiolytic activity in zebrafish larvae. Material and methods: In the first part of the experiment, the embryos were incubated with 5, 10, 20, and 40 mg/mL MI. 1-day post fertilization, embryo mobility was evaluated and burst activity was calculated. In the next part of the study, the behavior of 5-day-old larvae was tested. Results: Tests on embryo movement showed an increase in burst activity in the MI group at concentrations of 40 mg/mL (*p* < 0.0001) and a slight decrease in the group at concentrations of 10 mg/mL (*p* < 0.05). MI in the light/dark challenge had no impact on the thigmotaxis index. Conclusions: MI was shown to not affect stress reduction in zebrafish larvae. Further research on the potential of MI and other stereoisomers is needed.

## 1. Introduction

**Figure 1:**
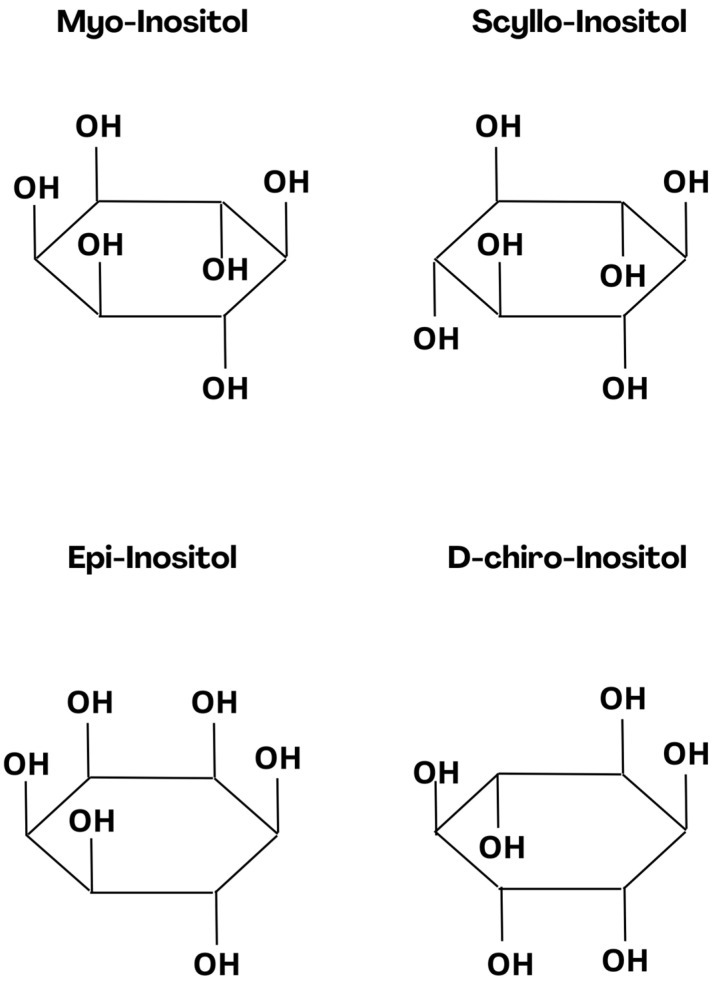
Chemical structure of Myo-Inositol and its derivatives with possible anxiolytic effect.

Herbal medicine and a healthy lifestyle based on a balanced diet full of fruits, vegetables, nuts and seeds have been promoted for years. Cyclitols are a group of compounds that may have a beneficial effect on human health and support immunity against numerous diseases, but its use as a clinical therapeutic is not established. Also known as sugar alcohols or polyols, they are widely distributed in the environment, and are found in many organic products such as citrus fruits, nuts, yeast, grains, beans, buckwheat, and many others [1]. Cyclitols and their derivatives perform many functions in eukaryotic cells. They regulate ion channel activity, intracellular phosphate storage, cell wall formation, signal transduction, membrane biogenesis and osmoregulation; furthermore, they are the forerunners of crucial secondary messengers [2]. Myo-inositol (MI) is a representative of the cyclitol group, at the same time being the most abundant inositol in nature. MI was first isolated from muscle extracts in 1850 by Scherer and since then has been intensively studied for its possible beneficial effects on the human body [3]. MI and its derivative supplementation are characterized by good drug tolerance, low toxicity, and possible use by pregnant women [4]. Figure 1 is depicting the chemical structures of MI and its derivatives with possible anxiolytic effects.

Since the start of the COVID-19 pandemic, the number of people suffering from depression and anxiety has increased substantially: a 27.6% increase in cases of major depressive disorders and a 25.6% increase in anxiety disorder cases [5]. Researchers have emphasized that it was a predictable phenomenon in the face of an unknown emerging crisis and that these numbers should normalize over time [6,7]. It is clear that during the pandemic onset, there was an acute increase in mental health symptoms [6]. The exact impact of the pandemic on the human mental condition needs to be determined by further longitudinal study [8]. The use of nutrient-based “nutraceuticals” and plant-based “phytoceuticals” to treat mental disorders is widespread. Still, no updated global clinical guidelines have been issued since 2015, until a recent meta-analysis by Sarris et al. where inositol was not advised for treating depression and lacked sufficient evidence for its effectiveness in treating anxiety [9]. Anxiety is one of the most widespread psychiatric disorders and it is still a significant problem among many patients suffering from other somatic diseases [10,11]. Currently used psychiatric drugs have many side effects and can lead to addiction. Natural methods of combating depression and drugs that would additionally carry a lower risk of complications are still being sought [12].

The best animal models for testing possible new treatment ideas are still being sought. Mouse models are expensive and require large quantities of samples, which is why an alternative is needed. For these reasons, the zebrafish (*Danio rerio*) has recently become the prominent model organism for research in many fields, such as toxicology, drug discovery, developmental biology, oncology, molecular genetics, and many others [13]. In the 1970s, George Streisinger from the University of Oregon was the first one to use the zebrafish as a model organism due to its low cost, the possibility of easier genetic manipulation than in mice models, and a fast development cycle. Zebrafish have many similarities in physiology and genetics to humans; moreover, 70% of disease genes function the same in humans and zebrafish [13]. Zebrafish enable high throughput screening and are compatible with the standard multi-well plates and video-recording systems used in industry [14].

The larvae of zebrafish are exceptionally applicable for behavioral tests performed in multi-well plates due to their small size and low-cost maintenance. Furthermore, these models are ideal for high throughput screening because of their relative maturity compared to adults in terms of swimming ability, motor functionality, sensitivity to stress, and their ability to perform simple motor tasks while receiving appropriate signals from the environment [14]. While the nervous system in zebrafish is less complex than in Homo sapiens, the action of the relevant neurotransmitters and adequate changes in their behavior in response to stress can still be observed. Researchers have used many tests to investigate zebrafish anxiety levels, such as the novel tank dive, open field test, light-dark test, startle test, electric shock assay, and others [15,16]. Thigmotaxis, also known as wall-hugging, is the tendency of the zebrafish to avoid the center of the arena and choose to stay near the edge of the well. Also, changes in the intensity of light affect the level of anxiety in zebrafish with the tendency to avoid the darkness. Such a behavior is commonly seen in nature in many species and demonstrates a peculiar reaction to stress [14,17].

The objective of our work was to examine the effect of MI on zebrafish embryos’ locomotor activity, as well as its potential anxiolytic activity in zebrafish larvae. 

## 2. Materials and Methods

### 2.1. The Fish Maintenance and Ethic Statement

All fish lines are housed in the fish facility of the Laboratory of Genomics and Transcriptomics, University of Warmia and Mazury in Olsztyn, Poland—built according to the local animal welfare standards. According to the European Directive 2010/63/EU and Polish legal regulations O.J. of 2015, item 266, studies performed on early-life-stage zebrafish larvae and euthanasia do not require Ethic Committee permissions.

### 2.2. Zebrafish Spawning, Embryo Selection, and Larvae Incubation

The adult AB zebrafish strain was set for spawning, with a female-to-male ratio of approximately 1:1, in spawning containers. The spawning was induced by turning on the light in the morning. Eggs in the stadium of 1 h post fertilization were collected and washed with E3 medium (5 mM NaCl, 0.17 mM KCL, 0.33 mM CaCl_2_-H_2_O, 0.33 mM MgCl_2_-6H_2_O, and pH 7.2). Collected embryos were selected and placed randomly in 5 Petri dishes with prepared solutions of MI (Chemat, Gdańsk, Poland). Four experimental groups were done consecutively: 5 mg/mL, 10 mg/mL, 20 mg/mL, and 40 mg/mL of MI diluted in E3 solution. The control group was incubated in the E3 medium. Prepared plates were incubated at 28.5 °C for 24 h. 

### 2.3. Embryo Movement Analysis

Tail coiling analysis was conducted on healthy 24 hpf embryos. Embryos in each treatment were selected to take 5-min videos under the stereomicroscope (SteREO Discovery.V8, Zeiss, Germany). Analysis was performed using DanioScope Software v 1.2.206 (Noldus, Wageningen, The Netherlands) [18]. Each group of embryos was recorded for 5 min. DanioScope automatedly analyses the video recordings of larvae and evaluates the parameters. Tail coiling activity was expressed as the proportion of the time of flicked tail in 1 min [%]. The proportion of the time of spontaneous head-tail contraction–burst activity [%] and total burst duration [s] of each embryo was counted and the average for each group was calculated.

### 2.4. Larvae Behavioural Assessment

All groups were incubated for 120 h. After every 24 h, solutions in all culture plates were changed. At 120 hpf, 24 larvae from each tested group were randomly chosen and transferred to 24-well plates (one individual larva per well) for behavioural tests. 

The measurements were performed using the DanioVision system with Ethovision XT v.15 software (Noldus, Wageningen, The Netherlands) [19,20]. The observation time for each larva was 25 min, of which the first 5 min were given for adaptation. The larvae spent the next 10 min in the light, and the last 10 min were spent in the dark. The distance moved [mm] was calculated for each larva.

**Figure 2:**
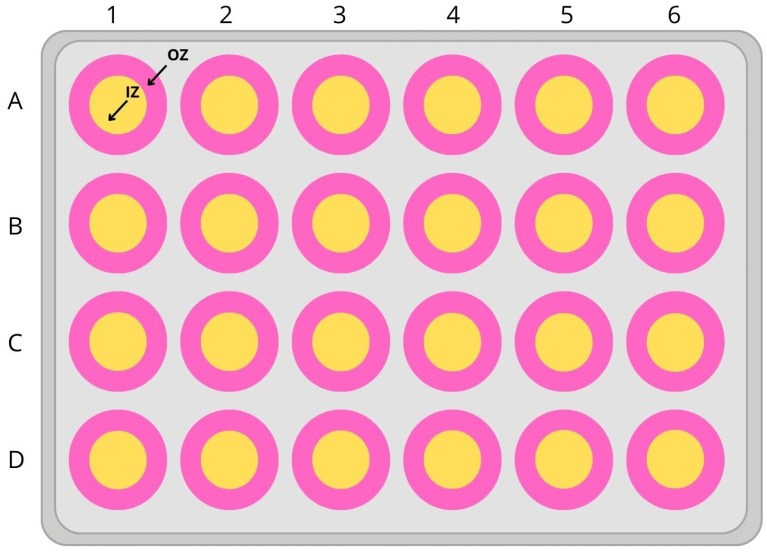
Schematic illustration of 24-well plate for larvae incubation (IZ—inner zone, OZ—outer zone).

To assess thigmotaxis, the swimming arena must provide adequate space for distinguishing between inner and outer zones. We used the 24-well plate format (diameter 16.2 mm). Figure 2 is a depiction of the schematic representation of how the inner and outer zones were defined.

The thigmotaxis was calculated as the ratio between total distance moved (TDM) in the outer zone of the test apparatus and TDM over the entire test arena (including inner and outer zones). The % of TDM in the outer zone was obtained by multiplying this ratio by 100 [14].

### 2.5. Statistical Analysis

All data were identified as normally or non-normally distributed using the Shapiro-Wilk test and expressed as a mean with standard error of the mean (SEM) to display more representative data. The quality of the two populations was tested using the Mann–Whitney U test. Multiple comparisons for comparing two or more independent samples between groups were performed using the Kruskal-Wallis test with Bonferroni correction. The data used to analyze the thigmotaxis index were cleaned by manually removing the extreme values (0% and 100%) and then removing outliers using the Grubbs test. *p* < 0.05 indicated statistical significance (ns: *p* > 0.05, *: *p* < 0.05, **: *p* < 0.01, ***: *p* < 0.001, ****: *p* < 0.0001). Statistical analyses were performed using R software (version 4.3.3) and the ‘ggstatsplot’ and ‘ggplot2’ approach.

## 3. Results

### 3.1. The Movement Analysis of Embryos 

**Figure 3:**
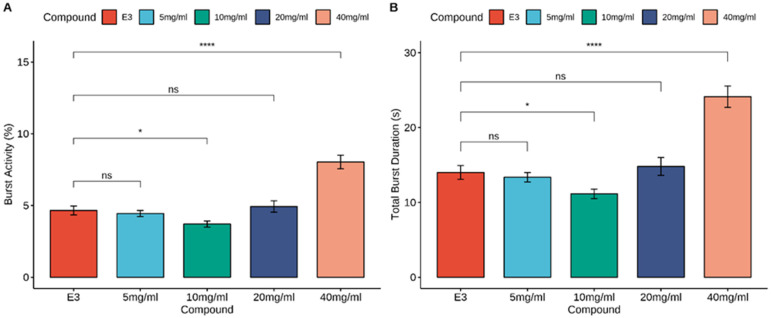
Effects of myoinositol (5, 10, 20, and 40 mg/mL) and E3 on locomotor activity. (**A**) Burst Activity. (**B**) Total Burst Duration. Data are presented as the mean ± SEM; n_E3_ = 39, n_5 mg/mL_ = 37, n_10 mg/mL_ = 37, n_20 mg/mL_ = 35, n_40 mg/mL_ = 37. ns > 0.05, * *p* < 0.05, **** *p* < 0.0001 n comparison to control group; (U Mann-Whitney test).

The Mann-Whitney U test showed an increase in burst activity in the control group and MI at concentrations of 40 mg/mL (*p* < 0.0001), and a slight decrease at concentrations of 10 mg/mL (*p* < 0.05). During the total burst duration analysis, a significant increase was observed when the larvae were treated with MI at a concentration of 40 mg/mL (*p* < 0.0001) compared to the E3-treated group. Additionally, a slight decrease in total burst duration was observed with MI at 10 mg/mL (*p* < 0.05). The results of zebrafish embryo locomotor activity are shown in Figure 3. 

### 3.2. Larvae Locomotor Analysis Using Dark-Light Test

#### 3.2.1. Larvae Locomotor Activity

**Figure 4:**
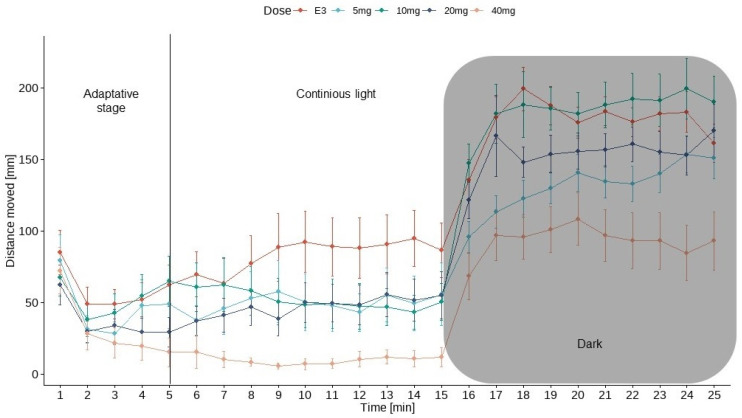
Light/dark challenge with larvae fish. Locomotor activity was recorded for 15 min before a sudden dark period lasting 10 min (gray shadowing). The fish were recorded for the entire 25 min; *n* = 24.

Figure 4 shows the results of locomotor activity testing under alternating light and dark conditions. Larvae were exposed to myo-inositol (5, 10, 20, and 40 mg/mL) or vehicle (E3 solution) for 25 min. Testing began in the adaptive stage (5 min), followed by one cycle of light (10 min) and darkness (10 min). Locomotion was not analyzed in the first stage. 

Data in Figure 4 are presented as the mean distance moved (in mm) and SEM in 1-min intervals during the 25-min sessions. For every group, the activity increased during the initial dark period (shaded area). 

**Figure 5:**
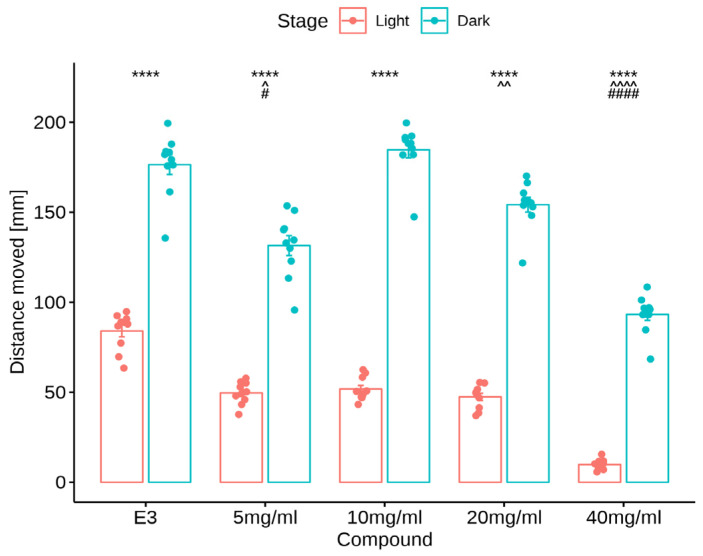
Effects of myoinositol (5, 10, 20, and 40 mg/mL) and E3 on locomotor activity. Average distance moved by zebrafish larvae within each 1-min time bin under either light (red bars) or dark (blue bars) conditions. Data are presented as mean ± SEM; n = 20. **** *p* < 0.0001 in comparison to light conditions within the same concentration group (U Mann-Whitney test); ^ *p* < 0.05, ^^ *p* < 0.01, ^^^^ *p* < 0.0001 in comparison to the control group under light conditions; # *p* < 0.05, #### *p* < 0.0001 in comparison to the control group under dark conditions (post hoc Bonferroni’s test).

The U Mann-Whitney test showed an increase in locomotor activity during the dark phase in the control group and MI at concentrations of 5 mg/mL, 10 mg/mL, 20 mg/mL, and 40 mg/mL (*p* < 0.0001) compared to the light phase. The Kruskal-Wallis test revealed statistically significant changes in zebrafish larvae behavior after incubation in myoinositol solutions in light [χ^2^(4) = 38.12, *p* < 0.0001] and dark [χ^2^(4) = 39.88, *p* < 0.0001] conditions. The post hoc Bonferroni’s test demonstrated a decrease in locomotor activity during the light phase in myoinositol at concentrations of 5 mg/mL (*p* < 0.05), 20 mg/mL (*p* < 0.01), and 40 mg/mL (*p* < 0.0001) compared to the control group (E3). During the dark phase of the experiment, a significant decrease in locomotor activity was observed after incubation in myoinositol at concentrations of 5 mg/mL (*p* < 0.05) and 40 mg/mL (*p* < 0.0001) compared to the E3 control group in the dark phase. The effects of MI and E3 on locomotor activity are shown in Figure 5.

#### 3.2.2. Thigmotaxis Index

**Figure 6:**
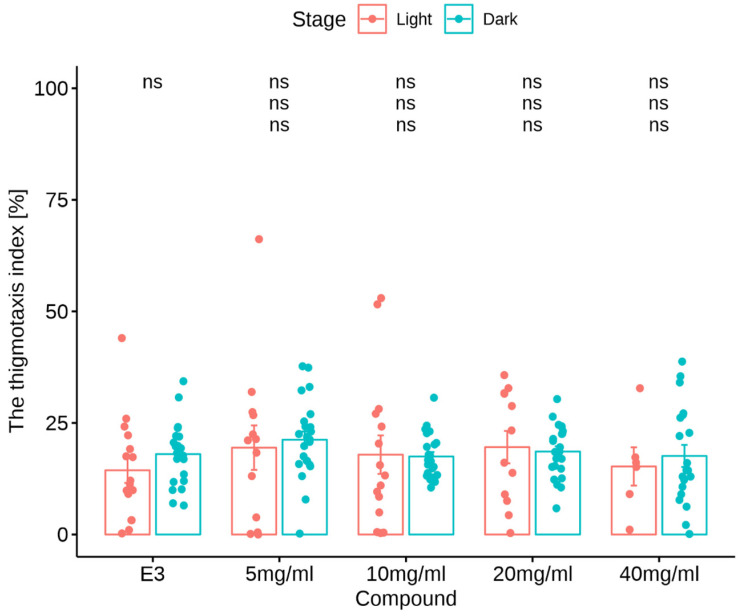
Effects of myoinositol (5, 10, 20, and 40 mg/mL) and E3 on thigmotaxis index measured as the % TDM in the outer zone. The thigmotaxis index by zebrafish larvae within each 1-min time bin under either light (blue bars) or dark (red bars) conditions. Data are presented as the mean ± SEM; ns (first upper row) *p* > 0.05 in comparison to light conditions within the same concentration group (U Mann-Whitney test); ns (second row) *p* < 0.05 in comparison to the control group under dark conditions; ns (third row) > 0.05, in comparison to the control group under light conditions (post hoc Bonferroni’s test).

Myoinositol treatment in the light/dark challenge had no impact on the thigmotactic behaviors of larvae (Kruskal-Wallis test: treatment under dark conditions [χ^2^(4) = 4.59, *p* = 0.33] nor was there an impact under light conditions (χ^2^(4) = 1.35, *p* = 0.85). The Mann-Whitney U test showed no change in the % TDM in the outer zone by zebrafish larvae after E3 (*p* > 0.05) and myoinositol treatments at concentrations of 5, 10, 20, and 40 mg/mL (*p* > 0.05) in the light phase of the experiment compared to the dark phase. During the dark challenge phase, no significant changes in the % TDM in the outer zone were observed when the larvae were treated with myoinositol at any concentration compared with the E3-treated group in the dark phase (*p* > 0.05). During the light phase of the experiment, no significant changes in the % TDM in the outer zone were observed when larvae were treated with myoinositol at any concentration, compared to the E3-treated group in the light phase (*p* > 0.05). The effects of MI on the thigmotaxis index are shown in Figure 6.

## 4. Discussion

Despite many years of research on the effects of MI, there are still no clear results of its use, and further research is needed to determine its effectiveness. It has already been speculated that supplementation with MI alleviates the insulin resistance symptoms in diabetic and Polycystic Ovary Syndrome (PCOS) patients, but the results of studies found in the literature are not clear. In some sources, we can find information about the MI’s ability to control insulin activity, improve insulin sensitivity, and has salutary effects in people with diabetes. Sharma et al. report that MI has the potential to regulate insulin, aiding in the prevention and management of diabetes mellitus [21]. Additionally, it has the advantage of being an inexpensive and safe alternative to commonly used drugs [22,23]. Unfer et al. performed an analysis of various studies on MI supplementation for improving hormonal imbalances in PCOS and provide the level Ia evidence of MI’s effectiveness [24]. In a study by Zhao et al. MI multicomponent supplementation with D-chiro-Inositol (DCI) was ranked best at improving menstrual frequency and this combination was found to be superior the supplementation of MI or DCI alone [25]. However, there are still few studies on the anxiolytic and antidepressant effects of MI. There are reports of reduced levels of brain MI in patients with depression and anxiety compared to healthy controls and low plasma MI concentrations have been suggested as a possible marker of major depressive disorders [26,27,28,29]. Also, PCOS patients often suffer from depression or anxiety, related to hormonal disturbances and weight gain. Cantelmi et al. report that MI supplementation turned out to reduce these symptoms [30]. In studies concerning possible antidepressant and anxiolytic inositol activity, researchers used MI and its stereoisomers such as Epi-Inositol (EI), Scyllo-Inositol (SI), and also D-pinitol; an analog of MI widely found in many plant families [30,31,32]. Most of these studies suggest the positive effect of MI supplementation on depression and anxiety [33]. However, as we mentioned before the recent guidelines by the World Federation of Societies of Biological Psychiatry (WFSBP) report that inositol is not advised for treating depression and lacks sufficient evidence for its effectiveness in treating anxiety Mashayekh-Amiri et al. [25]. Ref. [34] report an improvement in sleep quality in pregnant women supplemented with MI. SI contributes to the reduction of symptoms of depression and anxiety in patients with Alzheimer’s Disease [35]. EI is a stereoisomer absent in mammalian tissue, but present in pine bark. In research from Einat et al., the plus-maze model of anxiety in mice was used to test the anxiolytic-like activity of EI and MI. EI had the strongest anxiolytic-like effect, followed by MI, in comparison to the control group [36,37,38]. D-pinitol has also been shown to have an antidepressant and anxiolytic effect [39]. In a review of the plant-based methods of anxiety and depression treatment by Fajemiroye et al., researchers list a plant that originated in Brazil, *Mimosa pudica*, in which D-pinitol is one of the active principles. It has an antidepressant-like effect, mediated by the serotonergic system. It is used in the treatment of depression and insomnia and is consumed in the form of an infusion of dried leaves [40]. Alonso-Castro et al. analyzed the effects of an ethanol extract of *Senna septemtrionalis* aerial parts (consisting of 42% D-pinitol) on mice behavior. D-pinitol showed anxiolytic-like activity in the four anxiety models and this effect turned out to be stronger than after fluoxetine administration [39].

This is the second research to date in which MI activity was tested on the *Danio rerio*. In this study, we used concentrations of MI with a maximum value of 40 mg/mL. Based on our previous research, in which we conducted the fish embryo toxicity (FET) test, MI concentrations higher than 40 mg/mL turned out to be toxic and led to significant disturbances in zebrafish maturation and survivability [1]. In another research paper found in online databases, the authors investigated the impact of MI on stress reduction in another fish species—the turbot (*Scophthalmus maximus*). The researchers concluded that MI significantly reduces stress in individuals in response to salinity. MI was administered by dissolving it in the aquatic environment where the individuals lived and by adding it to their feed. The genetic testing of the transcriptome of the experimental group showed that MI increased the effectiveness of physiological processes such as steroid biosynthesis, steroid metabolism, circadian rhythm, tryptophan metabolism, metabolism of xenobiotics by cytochrome P450, oxidoreductase activity, iron ion binding, and heme binding [41]. 

The tail-coiling activity measurement performed via video recording of embryos is a relatively new alternative method for screening developmental neurotoxicity induced by tested substances. Tail coiling activity can be assessed based on many factors and one of them is burst activity; the percentage of time the embryo is moving in the recorded time bin [42,43]. In our work, burst activity of embryos was calculated and a significant increase in burst duration was observed at the highest dose of MI (40 mg/mL), but a minimal decrease in burst activity was detected in the 10 mg/mL group.

In this paper, we focused on determining the possible MI’s anxiolytic activity and behavioral changes in Zebrafish embryos and larvae. In the behavioral assessment, researchers often use the visual motor response (VMR) test which is based on a sudden transition in the illumination of the observed fish from light to darkness. Sudden cut-off of the light source causes a significant robust increase in the zebrafish locomotor activity due to the stress response [14,44]. In our study for every tested group, the activity increased during the initial part of the dark period, which is in line with other authors’ outcomes. Zebrafish larvae are showing aversion toward darkness, which leads to an increase of the traveled distance when compared to the light phase. In our research, average distance moved was higher in the dark than in the light conditions in every group. 

In the analysis of zebrafish larvae behavior, specific endpoints are used, such as total distance traveled, velocity, and time spent in zones, which allows for statistical data comparison [45]. In the papers concerning the anxiolytics tested on zebrafish, the total distance traveled by fish in combination with dark and light periods allows the assessment of anxiolytic or sedative effects. Zebrafish larvae show natural darkness avoidance (i.e., scotophobia) [46]. The results of our study remain ambiguous. While the characteristic zebrafish behaviors such as scotophobia remained unchanged, indicators showing changes in levels of anxiety were not significantly affected by MI. MI was found to reduce the distance moved in comparison to the control group. The main limitation of our study is that it is based on simple behavioral analysis. Our future studies will require biochemical validation as well as tracking the transcriptome changes in zebrafish.

Thigmotaxis is a validated index for the evaluation of anxiety changes in animals and humans. Thigmotaxis refers to the inclination to steer clear of the central area of an arena and instead remain close to or move along the boundaries of the surroundings [14,47]. The thigmotaxis index in experimental groups showed no significant changes than in the control group. Therefore, a significant anxiolytic effect of MI on zebrafish could not be demonstrated. 

## 5. Conclusions

Plant-based substances that can be used as potential anxiolytics are still being sought due to the many side effects of currently available drugs. In this project, we analyzed the effects of MI on zebrafish behavior, development, and potential anxiolytic effects. No direct anxiolytic effect of MI on zebrafish was demonstrated, but MI boosted the 24-hpf embryo locomotor activity and therefore it may have a beneficial role in the development of zebrafish embryos. It is worth noting that the group of cyclitols includes numerous derivatives, and dietary supplements containing MI are commonly used daily with remarkable effects. Still there are a few clinical trials registered, complited and with published results—20 studies to date about the effects of the whole inositol group, and none concerning the topic of depressive and anxiety disorders. Our research paves the way for further research on the action and effectiveness of MI and its other stereoisomers. Further studies are needed for a deeper understanding of the anxiolytic and developmental effects of MI, including transcriptome analysis using next-generation sequencing.

## Data Availability

Data available on request from the authors.

## Abbreviations

DCI	D-chiro-Inositol
EI	Epi-Inositol
FET	fish embryo toxicity
hpf	hours post fertilization
MI	Myo-Inositol
PCOS	Polycystic Ovary Syndrome
SI	Scyllo-Inositol
SEM	standard error of the mean
VMR	visual motor response
